# Effect of Sociodemographic Variables on Patient and Diagnostic Delay of Breast Cancer at the Foremost Health Care Institution in Nigeria

**DOI:** 10.1200/JGO.19.00108

**Published:** 2019-07-26

**Authors:** Sunday O. Olarewaju, Emmanuel O. Oyekunle, Adebukola O. Bamiro

**Affiliations:** ^1^ University of Osun, Osogbo, Nigeria; ^2^ University College Hospital, Ibadan, Nigeria; ^3^ Ladoke Akintola University of Technology, Ogbomosho, Nigeria

## Abstract

**PURPOSE:**

Breast cancer (BC**)** has the highest mortality rate among women with any form of cancer in developing countries. Unfortunately, patients with BC in Nigeria commonly present with late-stage disease. The current study examined the types and magnitude of delay in BC diagnosis at the foremost hospital in Nigeria and also identified the influencing factors.

**MATERIALS AND METHODS:**

This cross-sectional study involved questionnaires given to 275 patients with BC at University College Hospital, Ibadan, Nigeria, from August to October 2018. Sociodemographic characteristics and information relevant to management of their health problem were obtained after ethical committee approval. Data collected were analyzed by SPSS (version 23; SPSS, Chicago, IL) to assess the types and magnitude of delay experienced by patients, as well as identify related determinants using the appropriate statistical test with *P* = .05.

**RESULTS:**

The mean age of respondents was 49 (± 11.9) years with the majority being Yoruba (n = 154; 56%), Christians (n = 211; 76.7%), married (n = 193; 70.2%), employed (n = 151; 54.9%), having tertiary education (n = 142; 51.6%) and an average income of more than 18,000 naira (n = 176; 64%). Patient delay and diagnostic delay were observed among 97 respondents (35.3%) and 84 respondents (30.5%), respectively. Although patient delay was significantly associated with age, ethnicity, and marital status, the only variable significantly associated with diagnostic delay was marital status (*P* < .05). Level of income, education, employment status, and religion did not significantly (*P* > .05) contribute to either of these delays.

**CONCLUSION:**

BC management at the pioneer Nigerian teaching hospital is challenged by both delays in patient presentation at clinics and delays in the process of being diagnosed. Such delays need to be addressed to achieve favorable outcome of patients with BC in Nigeria.

## INTRODUCTION

Breast cancer (BC) causes 376,000 deaths a year, and approximately 900,000 to 1,000,000 women are diagnosed worldwide every year with the disease.^1^ It represents 18% to 28% of all cancers globally.^2,3^ BC has an incidence rate of approximately 40 per 100,000 women in Africa^4^ and also has the highest mortality rate among women with any forms of cancer in developing countries.^5^ In Nigerian women, it is the most common malignancy.^6,7^ The overall BC mortality rate in Nigeria is also considered to be relatively high. Whereas in America, 19% of all BCs result in death, this percentage is 51% in Nigeria, triple the rate seen in the United States.^8^ This indicates that less than 50% of Nigerian patients with BC survive for 5 years or longer after diagnosis. However, because figures for BC mortality are scarce,^9^ the true proportion of deaths could be significantly higher. The shocking increase seen in the 2016 cancer incidence data in Nigeria makes conducting studies regarding early diagnosis of BC in Nigeria crucial.^10^ Although BC is a major public health problem, early detection and diagnosis have shown promising results and a greater chance of survival of affected patients.^11^ Late diagnosis is a major factor for the high mortality seen in patients with BC from low- and middle-income countries because most patients present in the advanced stage of disease.^12,13^ In Nigeria and some sub-Saharan African countries, this is partly attributed to inadequate awareness and a lack of population-wide BC screening programs. Because BC is a topic that is not freely discussed in Nigeria because of cultural taboo, there is an urgent need for information and education on awareness of BC and its early detection measures. This information can help the health authorities to plan strategies for the early reporting of patients with BC to health service providers. A recent study^14^ showed that women in rural areas are the group most vulnerable to delay given that they are poorly informed and could be affected by lack of knowledge. This study can help close the gaps in this knowledge. Therefore, the aim of this study is to identify the types of delay before BC diagnosis and evaluate their related magnitude and determinant factors at University College Hospital (UCH), which is a national referral hospital.

**CONTEXT**

**Key Objective**
How do sociodemographic characteristics contribute to patient and diagnostic delay of breast cancer (BC) at the premier tertiary health care institution in Nigeria?
**Knowledge Generated**
Age and ethnicity are important determinants because they were found to have statistically significant associations with patient delay. However, marital status is considered the most important sociodemographic characteristic in this study because it contributes significantly to patient delay and is the only determinant also statistically associated with diagnostic delay.
**Relevance**
The findings in this study indicate a strong need for the government and nongovernmental organizations to increase literacy on BC management for adolescents and adults across the ethnic groups in Nigeria. More importantly, maximum social support should be solicited by all relevant organizations from family members of patients with BC, especially for married women. Efforts intensified in these directions to improve timeliness in BC management would enhance clinical outcomes.

## MATERIALS AND METHODS

This cross-sectional descriptive study involved questionnaires administered to 275 patients between August and October 2018 in the Department of Radiation Oncology, UCH, University of Ibadan, Ibadan, Nigeria. Approval for the study was granted by the UCH/University of Ibadan Ethics Committee. Verbal consent was obtained from the participants before the study. Eligible study patients included all female patients with BC who attended the surgical oncology and radiotherapy clinics at UCH during the study period.

The Leslie Fischer’s formula (n = Z^2^pq/d^2^, where p = 0.234 is the proportion of prevalence from a previous study,^14a^ CI is set at 95%, normal deviation Z = 1.96, d = 0.05, and q= 1− p) was used to calculate a minimum sample size of 275 participants for the study.

The data collection instrument used in this study was a self-designed questionnaire. Sociodemographic characteristics (SDCs) of the patients formed the first part of the questionnaire. The second part included symptoms of BC as noticed by the interviewees and their initial places of consultation. The third section addressed information on time intervals regarding seeking medical attention after noticing the earliest symptoms of BC (ie, patient delay [PD]). Although PD could range from less than 1 month, 1 to 3 months, or greater than 3 months, prolonged delays are usually defined as intervals greater than 12 weeks.^15^ Therefore, in this study, PD was considered to be a time lag of greater than 3 months because this is the most accepted threshold to establish delay.^16^ In addition, information regarding time to diagnosis was obtained. Time to diagnosis was considered to be the time from first date of reporting symptoms to a health care provider to the date of eventual diagnosis on the basis of medical investigations. Hence, diagnostic delay was defined in this study as a time interval exceeding 8 weeks between the two described dates. This threshold was previously used by McLaughlin et al.^17^ Open-ended questions were asked about the causes of delay, if any, in obtaining a medical consultation.

### Statistical Analyses

Data acquired were analyzed using SPSS version 20.0 (SPSS, Chicago, IL). The χ^2^ test was used to compare rates, ratios, and proportions, whereas the *t* test was used to determine the association between the continuous variables. The level of significance was set at *P* < .05.

## RESULTS

**FIG 1:**
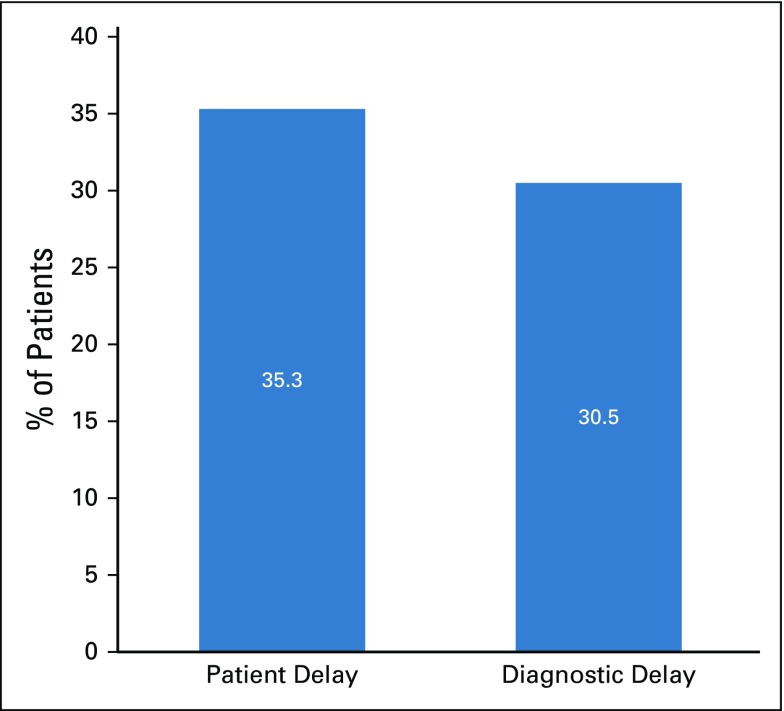
Proportion of respondents with patient and diagnostic delays of breast cancer at University College Hospital.

SDCs of the respondents are listed in Table 1. SDCs comprise seven variables that define the patients’ sociocultural status. Patients age 69 to 79 years constituted the peak age category and were the age group with the fewest patients. The proportion of single patients was small (< 5%). More than 60% of patients had a monthly income of greater than ₦18,000 (US$ 50), which was the national minimum wage at the time of the study. The symptoms (Table 2) experienced by the interviewees revealed that the majority of patients observed a painless mass that they would not have readily associated with breast malignancy. Although the majority of patients reported their symptoms first to medical doctors (Table 3), the promptness of patients reporting their symptoms was also of interest in this study. In Figure 1, the proportions of respondents who delayed reporting their symptoms are shown. Approximately 65% of patients reported their symptoms to medical personnel within 3 months, whereas approximately 70% had a definitive establishment of their diagnosis within 2 months of seeking medical consultation. Table 4 lists the causes of PDs and diagnostic delays as given by the interviewees. Nonawareness of the existence of BC was the least common cause identified for both types of delay. Marital status, age, and ethnicity were the SDCs found to be significantly associated with PD (Table 5). In Table 6, which lists the relationships between SDCs and diagnostic delays, marital status again stands out as the key variable.

**TABLE 1:**
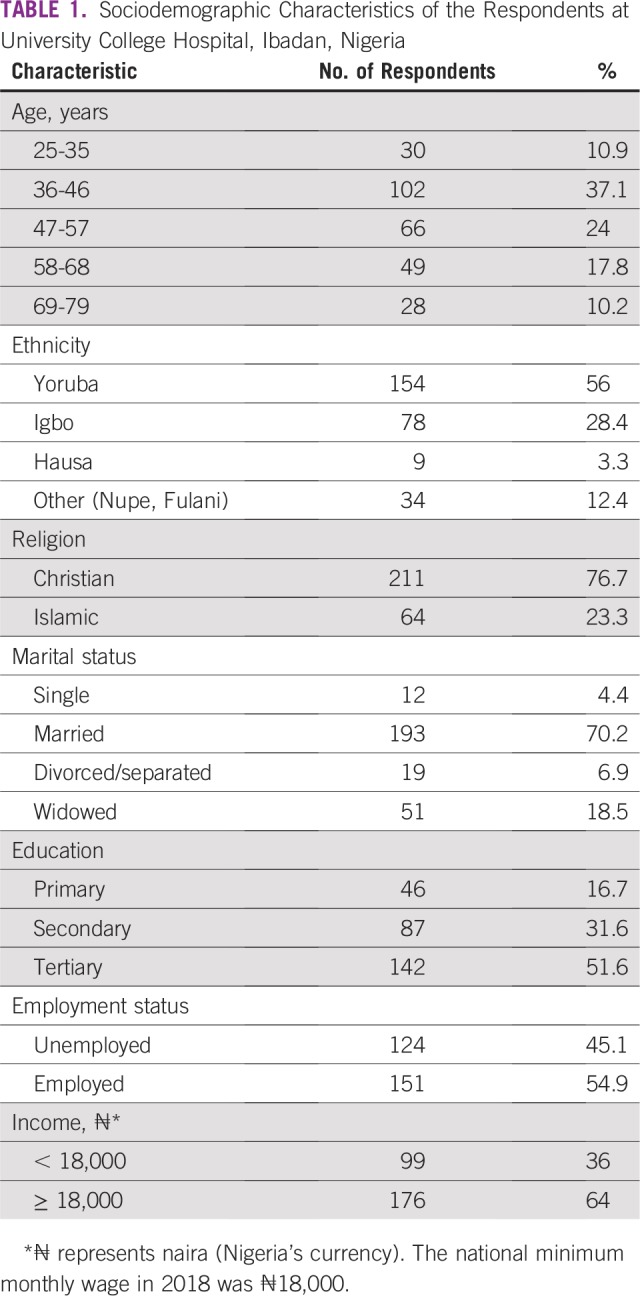
Sociodemographic Characteristics of the Respondents at University College Hospital, Ibadan, Nigeria

**TABLE 2:**
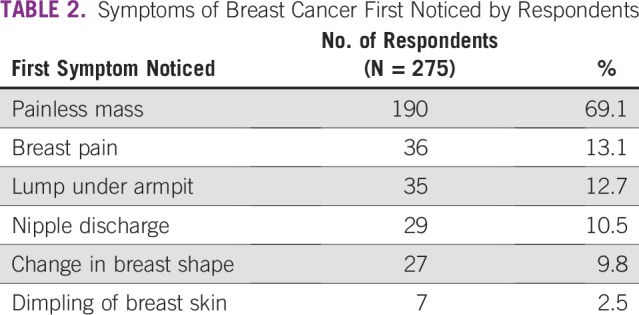
Symptoms of Breast Cancer First Noticed by Respondents

**TABLE 3:**
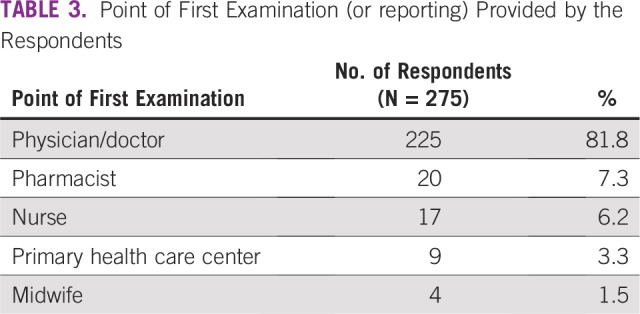
Point of First Examination (or reporting) Provided by the Respondents

**TABLE 4:**
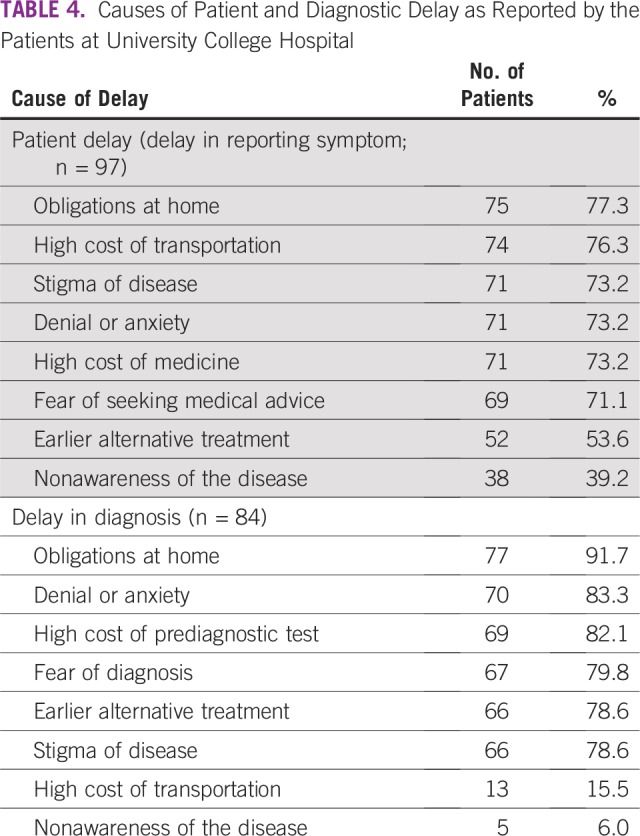
Causes of Patient and Diagnostic Delay as Reported by the Patients at University College Hospital

**TABLE 5:**
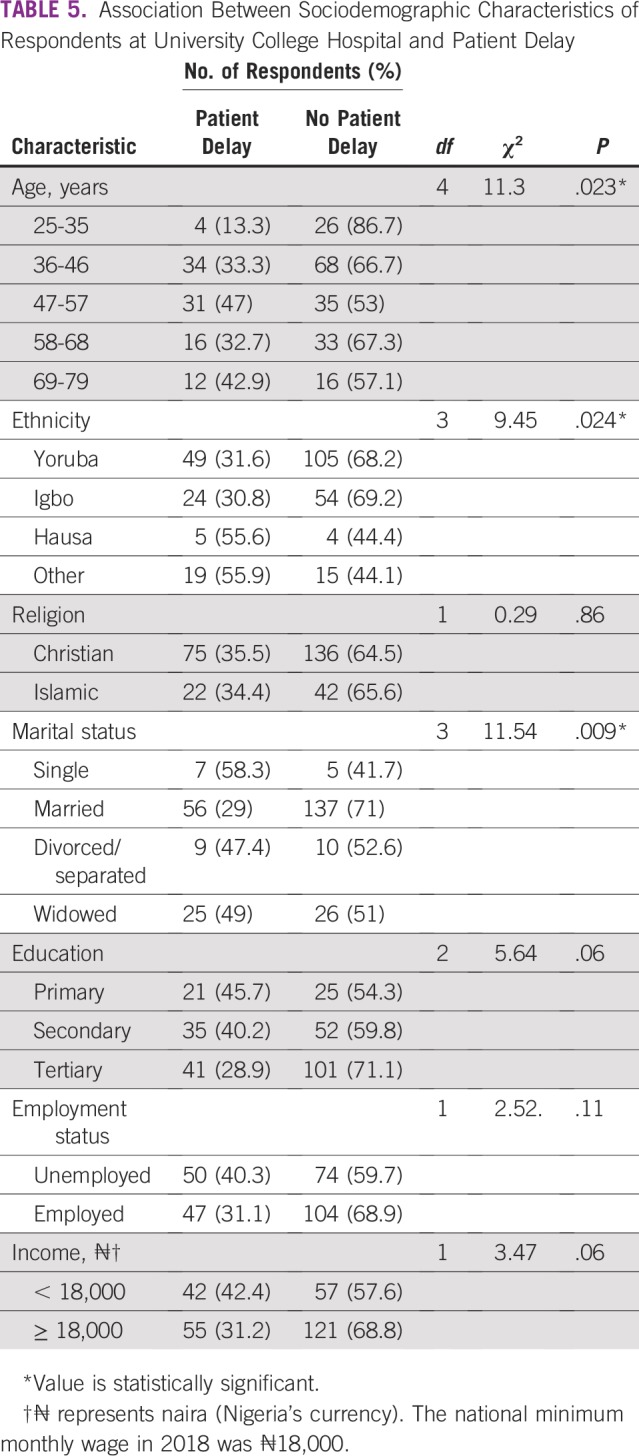
Association Between Sociodemographic Characteristics of Respondents at University College Hospital and Patient Delay

**TABLE 6:**
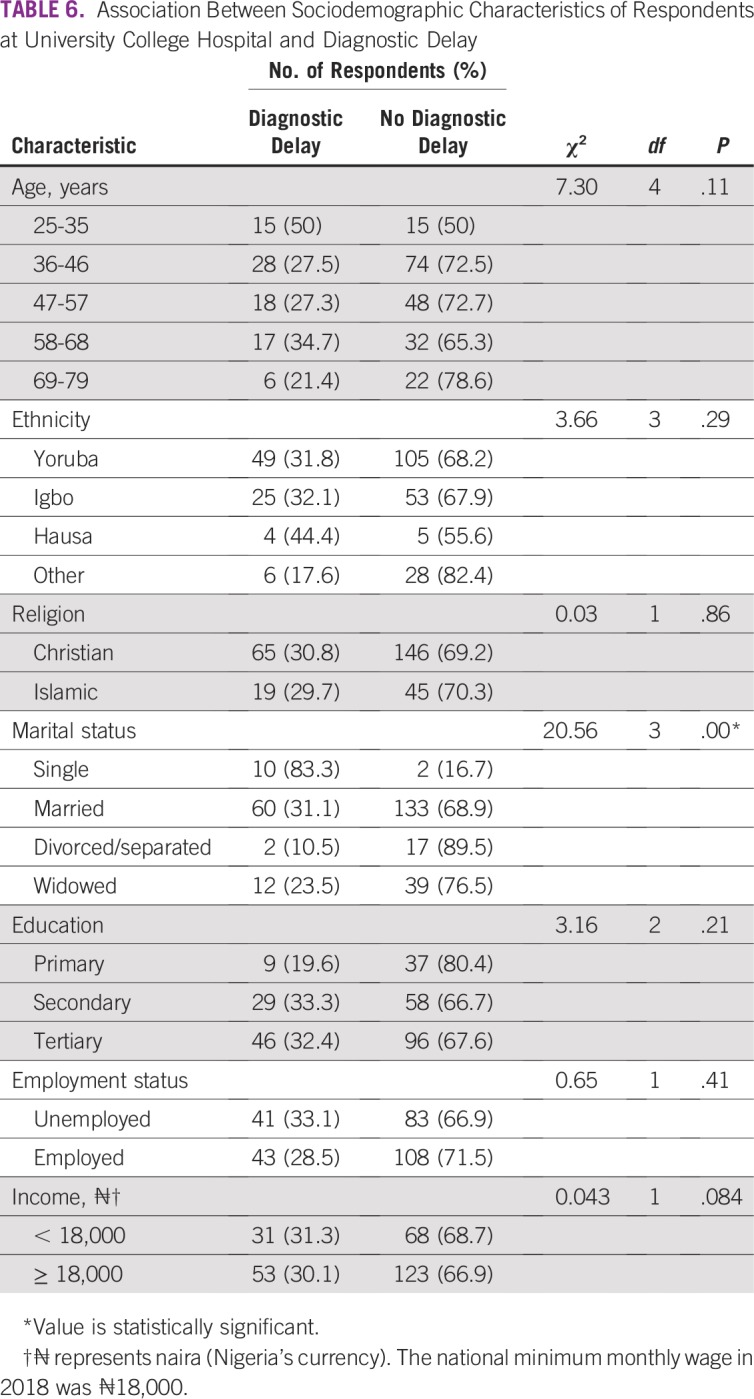
Association Between Sociodemographic Characteristics of Respondents at University College Hospital and Diagnostic Delay

## DISCUSSION

BC is the most commonly diagnosed cancer and the leading cause of cancer deaths in women worldwide, accounting for 23% of total cancers and 14% of all cancer-related deaths.^18^ In this study, the SDCs (Table 1) showed that the majority of the respondents falls between age 36 and 46 years, whereas the minority were between 69 and 79 years of age. The incidence of BC increases with age, whereas a younger age at diagnosis is linked to increased mortality.^19^ In Nigeria, where life expectancy is relatively low, it is feasible to find few adults older than age 65 years presenting in clinics for BC management. The life expectancy of Nigeria’s population, at 54.5 years, is one of the worst in the world according to the WHO.^20^

BC in women younger than age 35 years is uncommon and accounts for 2% of all BCs in the West.^21^ In a previous study of 320 Indian women with BC, 36 of the patients (11.3%) were younger than 35 years old.^21^ The proportion of women younger than age 36 years in the current study is comparable to that found in the study by Das et al,^21^ but both are much higher than the published Western data of 1% to 2%. The proportion of women living below the national minimum wage (36%) should not be overlooked because this could somewhat deter patients with BC from reporting their health conditions in a timely manner at the clinic.

A breast lump is the most common presenting symptom among women with BC and has relatively high predictive value for malignancy.^22,23^ In this study, a breast lump developed in 81.8% of the women (Table 2), which is comparable to the rate found in a previous study (83%).^24^ A breast lump is often initially painless, presenting as a swelling under the arm before the breast tumor is large enough to be felt. Less common signs and symptoms include breast pain or heaviness; persistent changes, such as swelling, thickening, or redness of the skin; and nipple abnormalities, such as spontaneous discharge (especially if bloody), erosion, or retraction.^25^ Prompt examination of the breast to investigate any of these changes is of high importance. Thus, the literature supports the current findings (Table 2), where a common symptom in many respondents was a painless mass, whereas less common symptoms included abnormal nipple discharge and breast pain.

Delayed patient presentation refers to a prolonged interval between discovery of initial symptoms and presentation to a provider and is typically defined as greater than 12 weeks, because delays of greater than 12 weeks have been associated with poorer survival.^26^ In this study, PD was found in more than one third of the patients, as shown in Figure 1. This finding is not unexpected because Table 1 indicates that larger percentages of patients were older than age 35 years, married, and educated. Therefore, many of the patients who were older and educated promptly reported their symptoms at the clinics. The delay of other patients in reporting symptoms could be largely attributed to their ignorance of subtle symptoms such as a painless lump. PD has been associated with increased tumor size, more advanced stage at presentation, and poorer long-term survival^27^ and is a significant concern in developing countries. Although the majority of patients (81.8%) consulted medical doctors first (Table 3), the proportion of patients who delayed presenting to a physician is still considerable and should not be overlooked

Another concern was the volume of patients who experienced diagnostic delay (Fig 1). As with PD, diagnostic delay was significant and is an issue to address if the likelihood of survival from BC would be enhanced by earlier diagnosis.

Table 4 lists the diverse causes of delayed patient presentation at clinics after development of symptoms and the causes of delays in diagnosis. The most important reason given for both types of delay concerns were the women’s obligations at home. This is logical given that the majority of the patients were married and had multiple responsibilities, including care of toddlers who would not have childcare if the woman were admitted to the hospital. These domestic demands were in addition to any educational and work demands, which could require ample attention. High transportation cost, which was considered by the women as a crucial reason for PD, can be explained by the fact that some patients covered distances of up to 400 km or more to arrive at UCH, which they deemed as the best location to address their health concerns. According to Table 1, a significant number of women came from tribes different from that found in the region of UCH. Therefore, there is no doubt that some patients had covered long distances to reach the foremost health institution. Also of high importance in the concerns expressed in Table 4 for PD is stigma regarding the disease. A diagnosis of cancer can be a stigma that reduces the self-esteem of individuals with the disease. Patients may be ashamed of having cancer and, more importantly, unwilling to hear about its progress. Other reasons for both PD and diagnostic delay (although to differing degrees) included denial or anxiety, high hospital bills, fear, earlier alternative treatment, and nonawareness of the disease. These causes of delay were related to certain SDCs, such as age, ethnicity, and marital status, which were found to have statistically significant associations with PD in this study (Table 5). Our findings show that more than 70% of patients who had PD or diagnostic delay identified fear as a cause of delay. Fear is an unpleasant emotion initiated by the threat of danger, pain, or harm. A previous review of factors contributing to late presentation of patients with BC in Africa showed that fear was a major factor. In Kenya, 19.9% of patients with BC presented late, mainly because of fear that they would be diagnosed with cancer.^28^ Heterogeneous statistics regarding fear were found in West Africa, ranging from 6.8% to 29.3% of patients in Nigeria^29-31^ to 34.8% of patient in Ghana.^32^ Thus, our single-center study presents a result consistent with these findings, with approximately 25% of the study population citing fear as a cause for delayed presentation or diagnosis. Conversely, in the United Kingdom, only 4.9% of patients with BC considered fear to be a reason for late presentation or delayed diagnosis.^33^ In most African countries, the disastrous nature of BC is intensified as a result of poor or limited resources, such as radiotherapy facilities, resulting in increased mortality rates.^34^ Symptoms of BC could thus trigger different kinds of anxiety, including fears of mastectomy, embarrassment, or divorce. Many Nigerians continue to use alternative or complementary treatment, and in our study, more than 50% of participants had PD or diagnostic delay as a result of seeking alternative treatment (Table 4). This finding supports previous work that identified the use of alternative medicine as a reason for women delaying seeking medical help and presenting late. The greatest use of alternative medicine was found in studies from West Africa.^28-33^ It should also be noted that nonawareness of BC was identified by approximately 40% of the respondents who experienced PD, which represents 13.8% of all study participants. A recent study^14^ done primarily among rural women in the southeastern part of Nigeria showed that approximately 30.4% of the study population lacked knowledge and awareness of BC. This value exceeds the related statistic we obtained (13.8%) because of the geographic area where the study was conducted and the low literacy level of the participants.

Reducing delays in care can significantly improve outcomes for patients with BC. In a previous study,^35^ patients with BC who experienced a short delay (< 3 months) experienced an absolute 7% greater likelihood of survival compared with patients who had moderate delays (3 to 6 months) in care. This magnitude of survival benefit was similar to or greater than the benefit achieved by chemotherapy. Thus, in this study, the proportion of patients with a delay of greater than 3 months in reporting their symptoms (51%) is of concern.

Barriers to seeking medical care may be related to financial constraints, geographic or transportation obstacles, nonavailability of services, and sociocultural or gender-related factors.^36^ One would expect some patients to be deterred from undergoing the process of diagnosis when female medical personnel are unavailable to oversee their clinical assessment.

At our institution, the breast clinic is open 2 days a week. Barriers within the health care system include inadequate facilities to cope with the high demand for medical care by patients with BC. Triple-modality assessment, including mammography, ultrasound, and biopsy, is often performed over a 2- to 3-week period of medical consultation because each test requires prior booking and, as such, is performed on separate days. This is a result of the large volume of people who need these investigations, resulting in considerable pressure on the hospital. Women who require mammography are often directed to a few private health care facilities around UCH to reduce their waiting times as the old mammography units at the institution are being considered for replacement. The average turnaround time for a biopsy report to determine whether oncology management is required is 2 weeks. In effect, people may wait for up to 5 weeks from medical consultation to find out whether they have BC. A time between presentation and diagnosis greater than 8 weeks was considered a diagnostic delay in this study, and therefore, the delays seen in the Nigerian health care system justified the present work.

Barriers within the health care system mainly include inadequate diagnostic and treatment facilities to cope with the high demand from the many patients with BC. Boycott of work as a result of disputes between health care workers and the government can be a major barrier to health care professionals providing timely care. However, this situation occurs only occasionally and was not the case for a considerable time before our study.

In this study, the only SDC strongly associated with diagnostic delay (Table 6) was marital status. This result is consistent with a previous study that showed that unmarried women were more likely to be diagnosed with BC at advanced stages. In addition, the previous study opined that many of the health benefits enjoyed by married women are likely derived from increased social support and social networks.^37^ Marital status is considered the most important determinant in this study because it contributes significantly to both types of delay (Tables 5 and 6). This finding is not farfetched. It could be duly inferred that the spouses of the patients with BC were involved in the health care decision-making process. Some of the patients who had work, business, or educational commitments likely lacked prompt support from family members or spouses. The stance on marital status is further supported by the predominant reason given for delay (Table 4), which was obligations at home.

This study is not without limitations. It is a single-center study, and the findings cannot be used to generalize the results to other situations in the country. Information on comorbidities and mode of transportation of study participants to UCH was not collected during data acquisition. However, the study achieved its primary aim and informed the need for related work in other parts of the country that would include additional parameters that were excluded.

In conclusion, BC management at UCH is challenged by both delays in initial patient presentation at clinics and delays in reaching medical diagnosis. Age, ethnicity, and marital status were found to have statistically significant (*P* < .05) association with PD. The study found no significant relationship (*P* > .5) between religion, education, employment, and income level and PD. Marital status was the only SDC found to contribute significantly (*P* < .05) to diagnosis delay, in addition to its influence on PD. It is recommended that similar studies are performed in the other five geopolitical zones of the country to further substantiate the effects of SDCs on delay in BC diagnosis in Nigeria.
